# Assessing the association between menstrual cycle phase and voice-gender categorization: no robust evidence for an association

**DOI:** 10.3389/fpsyg.2025.1531021

**Published:** 2025-04-02

**Authors:** Sarah Friedrich, Edward S. Brodkin, Birgit Derntl, Ute Habel, Philippa Hüpen

**Affiliations:** ^1^Department of Psychiatry, Psychotherapy and Psychosomatics, RWTH Aachen University Hospital, Aachen, Germany; ^2^Department of Psychiatry, Perelman School of Medicine at the University of Pennsylvania, Philadelphia, PA, United States; ^3^Department of Psychiatry and Psychotherapy, Tübingen Center for Mental Health (TüCMH), University of Tübingen, Tübingen, Germany; ^4^LEAD Graduate School and Research Network, University of Tübingen, Tübingen, Germany; ^5^Institute of Neuroscience and Medicine, JARA-Institute Brain Structure Function Relationship (INM 10), Research Center Jülich, Jülich, Germany

**Keywords:** menstrual cycle, voice-gender categorization, estradiol, progesterone, signal detection theory, mating cues, follicular phase, luteal phase

## Abstract

**Introduction:**

Hormone fluctuations during the menstrual cycle are known to influence a wide variety of cognitive-emotional processes and behavior. Mate choice and changes in attractiveness ratings for faces and voices are often investigated in this context, but research on changes in voice-gender perception independent of attractiveness ratings is rare even though the voice is an essential element in social interactions. For this reason, we investigated the influence of cycle phase and levels of estrogen and progesterone on performance in a voice-gender categorization task. Our expectation was to find a more pronounced other-sex effect, so faster and more accurate reactions for masculine voices, in the follicular (fertile) phase than in the luteal phase.

**Methods:**

We measured 65 healthy, naturally-cycling women, half of them in the follicular phase and the other half in the luteal phase. For the analyses, we used signal detection theory (SDT) measures in addition to reaction times and percent of correct reactions. The study was preregistered after measuring the first 33 participants and prior to any data analyses (https://osf.io/dteyn).

**Results:**

Cycle phase and hormone levels showed no significant effect on reaction time or SDT measures. This was the case both using frequentist analyses and Bayesian statistics. Reaction time was influenced by voice-gender, with faster reactions for feminine voices compared to masculine voices in both cycle phases.

**Discussion:**

Taken together, our results add to the increasing number of studies that do not find an interaction of menstrual cycle phase and reaction to gendered stimuli.

## 1 Introduction

The menstrual cycle, and associated changes in hormonal levels, have been shown to influence a variety of human functions, including cognition, emotion, physiology, and brain activity (Albert et al., 2015; Derntl et al., 2008; Derntl et al., 2013; Haraguchi et al., 2021; Hidalgo-Lopez et al., 2020; Pletzer et al., 2019). The primary hormonal fluctuations throughout the menstrual cycle involve changes in estradiol and progesterone levels. Both hormone levels are low during menses and the early follicular phase. In the later follicular phase, estradiol rises, peaking before ovulation which characterizes high fertility. After ovulation, the luteal phase begins, characterized by a rise in progesterone, which peaks mid-luteal phase, accompanied by a second estradiol increase. Finally, concentrations of both hormones begin to decline to their lowest levels during menses (Farage et al., 2008).

Associated with these hormonal fluctuations, changes in social behavior (Maner and Miller, 2014; Anderl et al., 2015) and mate selection (Puts et al., 2012) can be observed. For example, during the fertile phase of the cycle, women tend to prefer more masculine partners (Penton-Voak et al., 1999; Penton-Voak and Perrett, 2000) whereas during the nonfertile phase of the cycle, masculinity seems to be less important. Especially for faces, studies suggest a menstrual cycle related change in women’s preference with a higher preference for more masculine faces during the late follicular phase and during days prior to and directly after ovulation (Penton-Voak and Perrett, 2000; Johnston et al., 2001; Little et al., 2008). Evolutionary explanations suggest that higher levels of testosterone, which are associated with greater virility (Penton-Voak and Chen, 2004), may be associated with healthier offspring (Jones et al., 2008). This idea is supported by studies finding robust cycle shift effects for evaluation of potential short-term partners as opposed to only small or nul effects on choice of ponential long-term partners (Little et al., 2002; for an extensive review on cycle-shifts for attraction ratings see e.g., Gildersleeve et al., 2014 and Jones et al., 2008). Overall, the gender-categorization of human faces seems to be influenced by a variety of biological factors such as the viewers own gender role and sexual orientation (Luther et al., 2021), their age and experience with human faces (Hillairet de Boisferon et al., 2019) as well as previously activated categorial knowledge (Macrae et al., 2002).

Challenging the idea of a menstrual cycle-shift, a growing body of literature does not find a clear association between cycle phase and preferences for masculinized faces (Jones et al., 2018; Marcinkowska et al., 2016; Peters et al., 2009). Inconsistent findings may be related to methodological shortcomings such as inconsistent methods even within the same lab (Harris et al., 2013), low statistical power, and a lack of objective measures of cycle phase (Jones et al., 2019; Lewis, 2020). Another explanation for inconsistent findings is changes in participants’ visual processing (Garza and Byrd-Craven, 2019) and their visual discrimination abilities over the cycle. These changes have been demonstrated in increased visual sensitivity (Lewis, 2020; Parlee, 1983) and an increased ability to identify facial symmetry during fertile cycle phases (Lewis, 2017). Facial symmetry is generally interpreted as a sign of advantageous genetic traits and health (Fink and Penton-Voak, 2002; Foo et al., 2017), associated with a strong preference for symmetrical faces, independent of conscious detection (Little and Jones, 2006) thus substantiating the idea of evolutionary mating strategies exerting a strong influence on face perception and preference. Subconscious changes in the ability to identify facial symmetry or asymmetry may contribute to variations in face preference over the cycle. These hormonal variations not only affect preference and perception of faces, but also the appearance and attractiveness of women’s own faces. Through subtle changes in shape and skin structure, female faces are perceived as more attractive during the fertile phase (Bobst and Lobmaier, 2012; Puts et al., 2013).

While much of the literature focuses on changes in face preference during the menstrual cycle, less attention has been paid to other mating cues. One such cue is the human voice, which, like facial features, plays a key role for both mating (Hughes and Puts, 2021; Pisanski et al., 2018) and other social interactions (Guldner et al., 2020; Hellbernd and Sammler, 2016; McGettigan, 2015). The voice conveys pertinent characteristics which allow us not only to identify known voices, but also to characterize a stranger’s age, gender (Gallup and Frederick, 2010) or even health status (Arnocky et al., 2018). To do so, people rely mainly on two properties of the voice: the fundamental frequency (F0) and formant frequencies (Hillenbrand and Clark, 2009). The F0 is the average rate of vibration of the vocal folds per second and is closely related to the perceived overall pitch of a voice, while the formant frequencies are the result of the movement of the vocal apparatus during formatting vowels and consonants (Goldstein, 2014).

For women’s own voices, a robust association between menstrual cycle phase and voice quality can be seen. During phases of lower estradiol, the voice quality decreases in naturally cycling women, showing higher tension, roughness and instability (Arruda et al., 2019; Raj et al., 2010). Additionally, changes in F0 have been reported across the cycle, though with inconsistent direction (Bryant and Haselton, 2009; Fischer et al., 2011; Karthikeyan and Locke, 2015; Lã and Polo, 2020). However, research findings on changes in voice preference for male voices associated with the menstrual cycle are mixed. Whereas some studies find a clear inclination of women to more masculine voices during fertile cycle phases (Feinberg et al., 2006; Puts, 2005), other studies fail to find an effect of hormonal fluctuations on voice preferences (Jünger et al., 2018). These mixed results on the influence of the menstrual cycle on voice preference parallel the mixed results on face preference and perception.

However, studies on changes in voice perception over the cycle are still scarce. Nonetheless, this is an important factor in understanding the underlying mechanisms of potential preference changes, as illustrated by the previously described influences on face perception. Therefore, the goal of this study is to shed light on potential differences between cycle phases in voice-gender categorization as an important part of social interactions, where not only visual facial cues but also vocal information has to be integrated into a multisensory perception that guides behavior.

According to previous studies, similar to the perceived gender of faces, voice-gender categorization is influenced by both listeners’ gender (Junger et al., 2013; Smith et al., 2018) and sexual orientation (Smith et al., 2019). One way to study voice-gender perception is via a voice-gender categorization paradigm which uses words spoken by both natural male and female speakers alongside with voices morphed toward the opposite sex to investigate a person’s reaction to increasingly ambiguous stimuli. This kind of paradigm allows for the analysis of both the accuracy of responses and the response bias, meaning the inclination to a certain response in ambiguous situations. Overall, the aforementioned studies found an opposite sex effect for response accuracy in highly ambiguous trials together with a tendency for a response bias toward the opposite sex in those trials both heterosexual men and women (Smith et al., 2018; Junger et al., 2013; Junger et al., 2014). For both homosexual men and women however, response bias in ambiguous trials show a pattern more similar to heterosexual men, underlining an association of sexual orientation (Smith et al., 2019). Strikingly, the effect of listeners’ own sex on voice-gender categorization seems to be less robust in women than in men, as response patterns across studies show greater variance for women than they do for men. This may be related to hormonal fluctuations associated with the menstrual cycle. In fact, previous studies on voice-gender categorization have not taken the menstrual cycle phase into account (Smith et al., 2018).

Due to the high relevance of voices as cues for social interaction and mating and because of the influence of female sex hormones on mating cues, we first expected to find a behavioral difference between women in different cycle phases. Because the late follicular phase is associated with higher fertility and a greater preference for masculinity in heterosexual women, we expected a stronger other-sex effect in that cycle phase for response accuracy and reaction time. Secondly, we expected a difference in response bias, meaning the inclination to categorize a voice rather as masculine or feminine when the categorization is unclear. Specifically, we expected the response bias to be influenced by the estimated cost of a wrong decision. According to error management theory (Haselton and Buss, 2000), the response bias can be expected to be influenced by the estimated costs of incorrect decisions. The favored decision should be the one that results in the less costly error, so the smallest loss of resources, if the decision was wrong. Applied to female mate choice, it is unclear if the cost is higher for mistaking a male for a female and thereby missing a potential mate or if it is higher for mistaking a female for a male and thereby investing in a non-reproductive mate (Johnston et al., 2008). Thus, this is a more exploratory question, and we do not have prior assumptions for the direction of the difference. The influence of choice costs is expected to be more pronounced in the follicular phase since mating is more likely to result in offspring. Hence, we expect a stronger response bias in the follicular compared to the luteal phase.

## 2 Methods

All procedures were in accordance with the Declaration of Helsinki and were approved by the Independent Ethics Committee of the RWTH Aachen Faculty of Medicine. All participants gave written informed consent and received financial compensation of 10 €.

### 2.1 Participants

A total of 78 naturally cycling cisgender heterosexual women between 18 and 35 years (*M* = 25.48, *SD* = 4.11) participated in the study. Thirty-three of these datasets were collected in the context of an earlier study (unpublished data) but have not been analyzed before. The required sample size was 62 as calculated *a priori* using G*Power 3.1 (Faul et al., 2007). Based on the mixed results on associations of voice perception and cycle phase, as described in the introduction, we expected a small effect (Cohen’s *f* = 0.15) with a power of β = 0.80 and an α error probability of 0.05. In the context of mixed results and varying degrees of uncertainty, sample sizes in studies on the influence of cycle phase on perception and reaction to cues with mate value vary quite substantially, ranging from 50 or less (Oinonen and Mazmanian, 2007; Sanders and Wenmoth, 1998; Rosenberg and Park, 2002) to 200 or more (Jones et al., 2018; Jünger et al., 2018; Stern et al., 2021) and yield mixed results independent of sample size. Therefore, we decided to base our sample on the basic power calculation described above.

Participants were recruited using public flyers and online postings. All participants reported a regular menstrual cycle and did not take any contraceptives. Participants were recruited to be either in the follicular (*N* = 31) or the luteal (*N* = 34) phase (as determined by self-reports and hormonal profiles; see below) at the time of measurement. Only women whose reported menstrual cycle phase matched the cycle phase measured by blood samples were included. Consequently, 13 participants were be excluded from all analyses, because their self-reported cycle phase differed from the cycle phase determined, making a clear classification impossible. Both progesterone (*t*(63) = −8.25, *p* < 0.001) and estradiol (*t*(63) = −6.91, *p* = < 0.001) levels were significantly different between the two groups. There was no significant difference in age (*t*(63) = −1.45 *p* = 0.152) or years of education (*t*(62) = −1.29, *p* = 0.202) between groups. Demographic information and hormonal levels *(M ± SD)* are presented in Table 1.

**Table 1:** Final sample characteristics for both cycle phases.

	Follicular phase (*n* = 31) *M* ± *SD*	Luteal phase (*n* = 34) *M* ± *SD*	*p*
Age (years)	24.71 ± 3.04	26.18 ± 4.83	0.202
Education (years)	15.50 ± 2.68	16.33 ± 2.48	0.152
β-estradiol (pg/ml)	55.20 ± 89.0	130.9 ± 55.0	<0.001
Progesterone (ng/ml)	0.142 ± 0.08	9.565 ± 6.35	<0.001

Prior to enrolment, each woman took part in a telephone interview to assess eligibility for the study and to assess the current day of the cycle. Exclusion criteria were hearing or speech impairment, use of oral contraceptives or other hormones, diseases or medications known to affect the endocrine system, pregnancy, or breastfeeding, and neurological or mental disorders. Physical illness, medication and pregnancy were assessed by self-report. The absence of mental disorders was assessed using the clinical version of the structured clinical interview for DSM-5 (SCID-5 CV; First et al., 2016). One participant had to be excluded due to an assumed presence of a mental illness based on the SCID interview. To control for a potential influence of sexual orientation, only heterosexual women were included in this study. Sexual orientation was assessed via self-report.

### 2.2 Cycle phase determination

To determine the cycle phase, participants were asked for the first day of their last menses during the telephone interview. To schedule the measurement date, they had to inform the study team via email as soon as their next menses started. If the time frame between both menses fell into a regular cycle (23–35 days), participants were randomly assigned to either the follicular phase group (7–11 days after onset of the current menses) or the luteal phase group (17–34 days after onset of the current menses). Since the cycle phase is often inaccurately self-reported (Farrar et al., 2015), we confirmed the estimated cycle phase by assessing levels of progesterone (P) and estradiol (E) via blood serum samples, assessed using ElektroChemiLumineszenz-ImmunoAssays (ECLIAs). The reference range for estradiol was 20.5–233 pg./mL for the follicular and 30.2–305 pg./mL for the luteal phase. For progesterone, the reference range for the follicular phase was <0.05–0.323 ng/mL and for the luteal phase 0.537–20.9 ng/mL. Progesterone levels below the detection limit (0.05 ng/mL) were entered as half the detection limit (0.025 ng/mL). In our sample, this was the case for 4 women, all of them in the follicular phase. For higher reliability of cycle phase determination, people not involved in data collection or analyses rated the cycle phase for each woman based on hormonal levels according to reference ranges. Additional information about the procedure can be found in the Supplementary information 1. This combined approach of forward counting of cycle days and assessing the level of reproductive hormones allows us to substantially reduce the uncertainty of true cycle phase for each participant. This procedure enables detecting effects with a much smaller sample size than usually required in studies using counting methods alone (Gangestad et al., 2016; Jonge et al., 2019; Maki et al., 2002).

### 2.3 Procedure and paradigm

At the beginning of the session, each woman completed a short interview including questions on demographic data, current cycle phase and exclusion of hormone intake. This interview was followed by a screening version of the SCID-5 to exclude mental disorders, and a blood withdrawal (ca. 7 mL) to assess blood serum hormone levels. Due to practical reasons, we could not control for time of day for the blood withdrawal. Subsequently, participants completed a voice-gender categorization paradigm. An extensive description of the paradigm can be found in Junger et al. (2013). The stimuli consisted of 6 trisyllabic, neutral nouns, each spoken by 5 male and 5 female speakers. The resulting 60 words were each morphed 2, 4 and 6 semitones (st) toward the speaker’s other sex by adjusting the pitch contour and the formant structure as a reflection of vocal tract length accordingly using the “change gender” function implemented in the software Praat Version 5.2.03 (Boersma and Weenink, 2010). These final 240 words were presented pseudorandomized in a way that no speaker and no word was presented consecutively. The presentation was divided into 80 blocks, each consisting of 3 words spoken by the same sex and morphed to the same degree. The stimuli were delivered via headphones using the software Presentation Version 21.1 (Neurobehavioral Systems, Inc., 2019). Participants were instructed to categorize the speaker’s sex for each stimulus as male or female as fast as possible by pressing the number key “7” for male speakers and number key “8” for female speakers on a laptop keyboard.

### 2.4 Data analyses

Behavioral data were analyzed using Matlab2019a (MathWorks, Inc., 2019). Sociodemographics, group differences and correlations were analyzed using the software R version 4.1.2 (RStudio Team, 2021). The study has been preregistered after measuring the first 33 participants and prior to any data analyses.^1^

#### 2.4.1 Between- and within-group behavioral differences

Reaction time differences were calculated using three mixed-model ANOVAs, with overall reaction time, reaction time for correct trials and reaction time for incorrect trials as dependent variables, respectively. The models contained the between-subjects factor cycle phase (follicular vs. luteal phase) and the two within-subject factors voice-gender (masculine vs. feminine) and morphing level (0, 2, 4, or 6 st morphing) as independent variables. For significant effects, post-hoc pairwise comparisons were calculated. All post-hoc comparisons were Bonferroni-corrected to account for multiple testing. Effect sizes were calculated using generalized eta squared.

The frequentist approach to statistics has been increasingly criticized (Jarosz and Wiley, 2014). Major points of criticism have been the arbitrariness of a *p*-value of 0.05 as a cut-off as well the influence of sampling and sample size on the p-value, which can lead to significant results, that are only valid within the given sample (for an extensive overview, refer to Wagenmakers, 2007). Therefore, we decided to validate effects of cycle phase using Bayesian ANOVAs to assess the likelihood for H_0_ (cycle phase does not influence task performance) over H_1_ (there is a significant difference between cycle phases) using the Bayes factor (BF_01_). Conventions for interpreting the resulting BF are provided by Raftery (1995) and define a BF between 1 and 3 as weak, between 3 and 20 as positive, between 20 and 150 as strong and larger than 150 as very strong. Bayesian analyses were conducted using the BayesFactor package for R. For all models 10,000 iterations were run, participants were included as a random factor.

#### 2.4.2 Signal detection theory

To allow for a more detailed investigation of the underlying mechanisms of potential performance differences, we employed signal detection theory (SDT) measures. The SDT is a well-established model for decision making processes (Lynn and Barrett, 2014; Stanislaw and Todorov, 1999). It was originally developed for signal vs. noise psychophysical perception tasks (Green and Swets, 1974) and differentiates between discriminability (i.e., ability to detect a target stimulus from background events) and a response bias (i.e., a tendency toward a certain response independent of the stimulus) (Stanislaw and Todorov, 1999). SDT measures have been employed in previous studies using the same voice-gender categorization paradigm and proved to be suitable for detecting differences in gender categorization ability (Junger et al., 2013; Smith et al., 2018; Smith et al., 2019; Junger et al., 2014). To calculate SDT measures for our paradigm, male voices were defined as target and female voices were defined as noise. This definition is arbitrary and chosen to match previous studies ability (Junger et al., 2013; Smith et al., 2018; Smith et al., 2019; Junger et al., 2014). This results in a definition of correct reactions to male voices as hits and incorrect reactions to male voices as misses. For female voices, correct reactions are defined as correct rejections, while incorrect reactions are defined as false alarms.

Since the Shapiro–Wilk test revealed deviances from normality of the data, we used non-parametrical measures. These measures were A′ for discriminability (Equation 1) and B″_D_ for response bias (Equation 2) and can be calculated using the following formulas (Pallier, 2002):


(1) $$\text{If hits}\;>\;\text{false alarms:}\;A^{\prime }=\frac{1}{2}+\frac{\left(\mathrm{hit}-fa\right)*\left(1+\mathrm{hit}-fa\right)}{4*\mathrm{hit}*\left(1-fa\right)}$$

$$\text{If false alarms}\;>\;\text{hits:}\;A^{\prime }=\frac{1}{2}+\frac{\left(fa-\mathrm{hit}\right)*\left(1+fa-\mathrm{hit}\right)}{4*fa*\left(1-\mathrm{hit}\right)}$$

(2) $$B{\prime \prime }_{D}=\frac{\left(1-\mathrm{hit}\right)*\left(1-fa\right)-\mathrm{hit}*fa}{\left(1-\mathrm{hit}\right)*\left(1-fa\right)+\mathrm{hit}*fa}$$

A′ ranges from 0 to 1 with higher values indicating better discriminability (i.e., a high rate of correct reactions to both male and female speakers) and values near 0.5 indicating performance on chance level (Pallier, 2002). B″_D_ ranges from −1 to 1 with a value of 0 indicating no response bias, positive values indicating in this case a tendency to categorize a voice as feminine and negative values indicating a tendency to categorize a voice as masculine. As each trial must be categorized as either a hit/correct rejection or a miss/false alarm for SDT analysis, non-response trials were not considered.

To test for differences between groups and conditions, three mixed-model ANOVAs were performed with cycle phase (follicular vs. luteal phase) as between-subjects factor and morphing level (0, 2, 4, 6) as within-subject factor. A′ was the dependent variable for the first ANOVA, whereas B″_D_ was the dependent variable for the second. Additional to the SDT measures, a mixed-model ANOVA with percent of correct responses as dependent variable was calculated. While the normality assumption was not fulfilled, homoscedasticity was given, therefore the results of a mixed-model ANOVA can still be assumed to be robust despite the non-parametric distribution (Harwell et al., 1992). For all variables where Mauchly test indicated a lack of sphericity, degrees of freedom were corrected using Greenhouse–Geisser adjustment (Greenhouse and Geisser, 1959).

#### 2.4.3 Signal detection theory and reaction times

One of the major drawbacks of the SDT is that it does not take RT into account. Therefore, we assessed potential associations between SDT values and RT in both cycle phases using correlations. The Shapiro–Wilk-Test indicated a lack of normal distribution for SDT values (*p* > 0.05), so Kendall’s *τ* was chosen as a measure of correlation. SDT measures and mean reaction times for male and female speakers were correlated over all morphing levels. To control for multiple testing, Holm correction was used (Holm, 1979).

#### 2.4.4 Influences of morphing, speaker, and hormone levels on performance

To allow us to look at hormonal influences and their interactions with morphing level and the voice-gender regardless of cycle phase, we ran multiple regressions with response bias B″_D_ and with RT as dependent variables and hormone levels (P and E) as independent variables. This approach gave us the possibility to include hormone levels as a continuous variable as regression analyses have a higher statistical power than ANOVAs. To identify influential predictor variables, we compared increasingly complex models by stepwise adding predictors and interactions. For both B″_D_ and RT we started with simple models including only variables as determined by the design of the paradigm: we started with morphing level, followed by voice-gender as a predictor for RT. Finally, hormone levels were added as further predictors. Additive models as well as interactions were tested. Since our previous analyses did not yield a significant difference between the two cycle groups, we did not include cycle phase as a factor. Model fits were compared based on Akaike information criterion (AIC) and Bayesian information criterion (BIC) values. AIC and BIC are both calculated using a model’s maximum likelihood estimate and correcting for number of model parameters (Vrieze, 2012). A major difference between both criteria is that BIC is growing more restrictive with an increasing number of parameters. Thus, BIC is more consistent, as long as the true model has a finite number of parameters and is one of the models, that are tested. In cases, where the model is more complex, the AIC is preferred (Vrieze, 2012). Due to the different calculations, we included both measures in our model selection. Additionally, we tested for significant differences between the models using Chi-Square test.

## 3 Results

### 3.1 Reaction time

For overall RT, we found significant main effects of voice-gender (*F*(1, 63) = 44.18, *p* < 0.0001, η^2^ = 0.023) and morphing level (*F*(3, 189) = 206.38, *p* < 0.0001, η^2^ = 0.193) as well as an interaction for both variables (*F*(3, 189) = 39.96, *p* < 0.0001, η^2^ = 0.022), but no main effect for cycle phase (*F*(1, 64) = 2.15, *p* = 0.148). Post-hoc comparisons showed lower reaction times for feminine voices compared to male voices (*t*(259) = −8.20, *p* < 0.001). For morphing level, we found significant RT differences for each morphing level compared to another (all *p* < 0.001). Post-hoc tests for interaction effects of voice-gender and morphing level showed a significant influence of voice-gender on RT for each morphing level with faster RTs for feminine voices for 4 st (*F*(1, 64) = 52.32, *p* < 0.0001, η^2^ = 0.065) and 6 st morphing (*F*(1, 64) = 100.94, *p* < 0.0001, η^2^ = 0.085).

**Figure 1:**
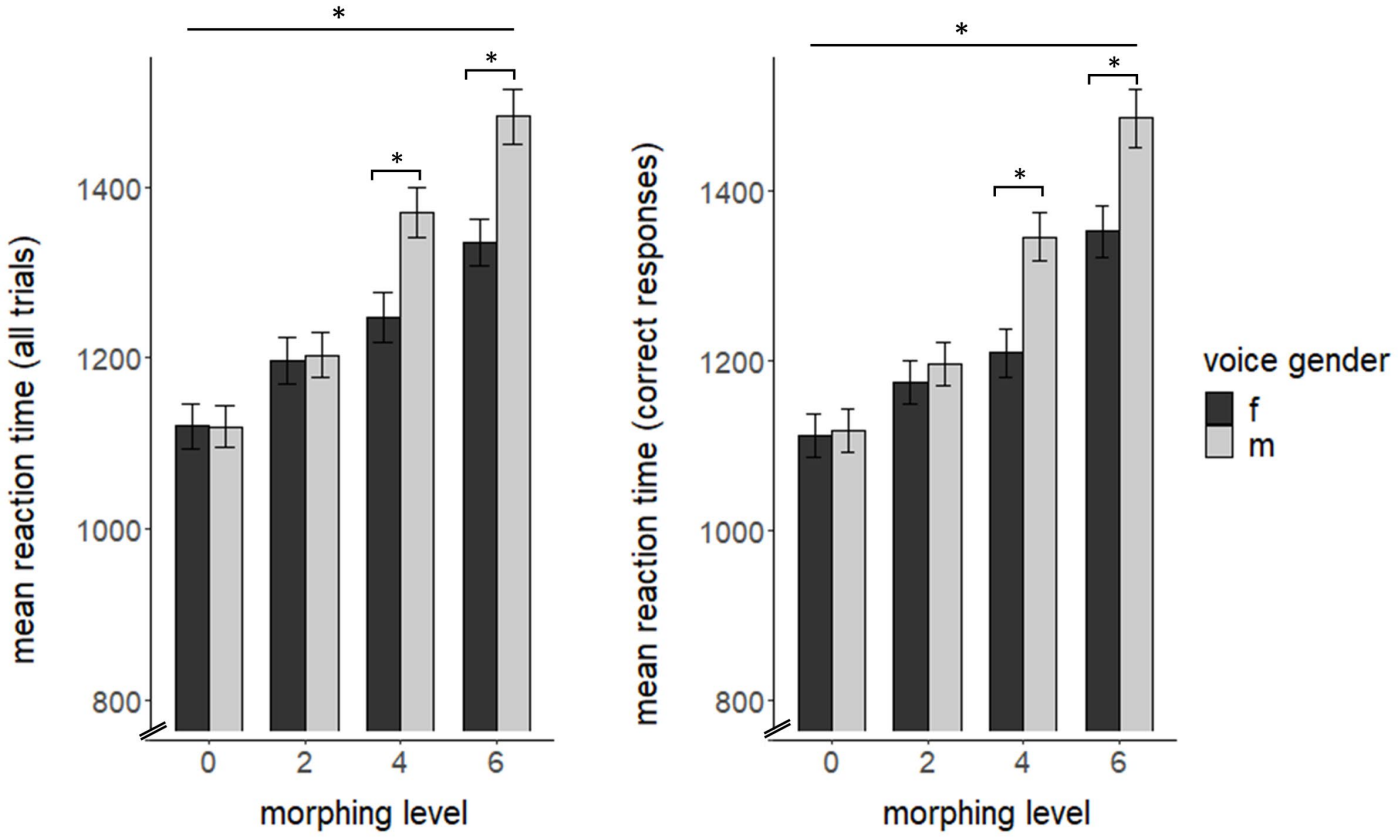
Reaction time as a function of morphing level and voice gender for all trials (left) and for correct reactions (right) across cycle phase. f = female speaker, m = male speaker. Reaction time is depicted in ms. The asterisk on the upper line indicates significant differences between all morphing levels. Results of the pairwise comparisons are listed in the Supplementary material.

Comparable to overall RT, RT for correct trials also showed significant effects of voice-gender (*F*(1, 63) = 38.67, *p* < 0.0001, η^2^ = 0.027) and morphing level (*F*(3, 189) = 231.88, *p* < 0.0001, η^2^ = 0.204) as well as an interaction for both variables (*F*(3, 189) = 27.1, *p* < 0.0001, η^2^ = 0.018). Again, post-hoc pairwise comparisons showed faster RT for feminine voices over all morphing levels (*t*(259) = −8.21, *p* < 0.001), significant differences between each morphing level (all p < 0.001) and a significant interaction of voice-gender and morphing level for 4 st (*F*(1, 64) = 69.33, *p* < 0.001, η^2^ = 0.082) and 6 st (*F*(1, 64) = 33.28, *p* < 0.001, η^2^ = 0.061) morphing (see Figure 1) with faster RT for feminine voices. We did not find any significant RT effects of incorrect reaction trials (all *p* > 0.05).

To substantiate the null effect for menstrual cycle phase, we conducted a Bayesian ANOVA. Results for overall RT (BF_01_ = 1.975), RT for correct responses (BF_01_ = 2.038) and RT for incorrect responses (BF_01_ = 2.922) supported the H_0_, though only to a weak extent.

### 3.2 Signal detection theory—discriminability and response bias

**Figure 2:**
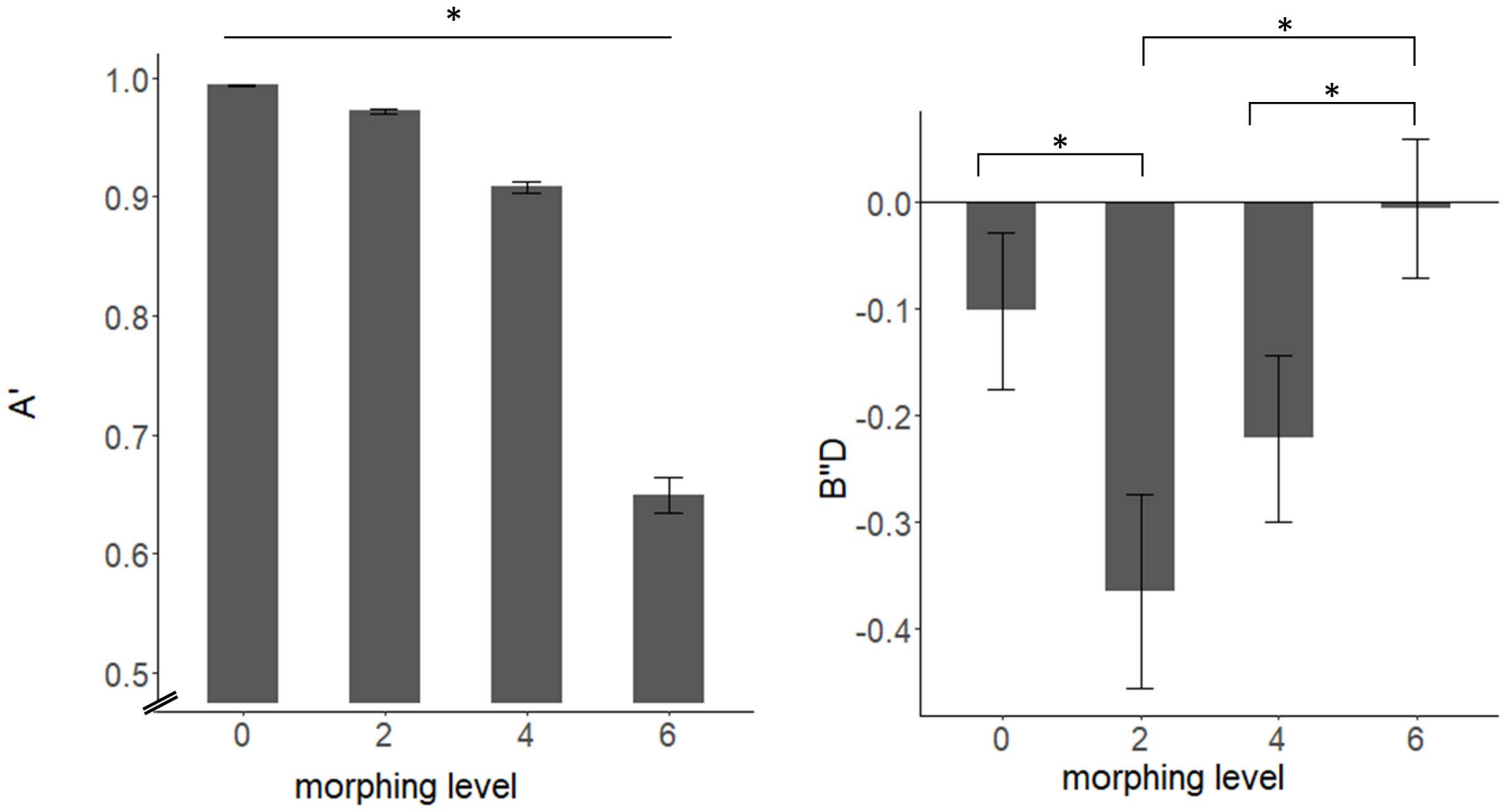
Differences in discriminability A’ (left) and response bias B’’D (right) for different morphing levels. The asterisk on the upper line indicates significant differences between all morphing levels.

Results of the mixed-model ANOVA revealed a significant main effect of morphing level for both discriminability A′ (*F*(1.17, 73.65) = 460.9, *p* < 0.001, η^2^ = 0.829) and response bias B″_D_ (*F*(2.27, 143.17) = 9.43, *p* < 0.001, η^2^ = 0.046). Pairwise comparisons showed significant differences between all morphing levels (see Figure 2) with decreased values for A′ for higher morphing levels (all *p* < 0.001). Pairwise comparisons for B″_D_ showed differences for 0 compared to 2st morph (*t*(64) = 2.88, *p* = 0.033), 2st to 6st morph (*t*(64) = −5.03, *p* < 0.001) and 4 st to 6 st morph (*t*(64) = −5.35, *p* < 0.001). Results of the pairwise comparisons are listed in the Supplementary material.

There were no significant main effects of cycle phase (*F*_A’_(1, 63) = 2.55, *p*_A’_ = 0.115, η^2^ = 0.013; *F*_B″D_ (1, 63) = 0.24, *p*_B″D_ = 0.625, η^2^ = 0.003) and no interaction effects of cycle phase and morphing level (*F*_A’_(1.17, 73.65) = 2.02, *p*_A’_ = 0.157, η^2^ = 0.021; *F*_B″D_ (2.27, 143.17) = 1.04, *p*_B″D_ = 0.364, η^2^ = 0.005).

In line with that finding, Bayesian ANOVA results were in favor of the H_0_ for both A′ (BF_01_ = 5.263) and B_″D_ (BF_01_ = 3.124).

In line with SDT measures, for percent of correct answers we found a main effect for morphing level (*F*(1.87, 112.14) = 998.60, *p* < 0.001, η^2^ = 0.679), but no effect for voice-gender or cycle phase and no interaction effects (all *p* > 0.05). Bayesian ANOVAs also favored H_0_ regarding effects of menstrual cycle phase (BF_01_ = 7.136). Post-hoc tests showed significant differences in percent of correct answers for each morphing level compared to another (all *p* < 0.001).

### 3.3 Signal detection theory and reaction times

Within the follicular phase group, we found negative correlations between A′ and mean RT for both feminine (*r_τ_* = −0.21, *p* = 0.003) and masculine (*r_τ_* = −0.35, *p* < 0.001) voices. In the luteal phase we also found negative correlations between A’ and mean RT for feminine (*r_τ_* = −0.26, *p* < 0.001) and masculine (*r_τ_* = −0.35, *p* < 0.001) voices. Additionally, we found a positive correlation between B″_D_ and mean RT for masculine voices (*r_τ_* = 0.20, *p* = 0.003) that did not become apparent in the follicular phase (*r_τ_* = 0.06, *p* = 0.545).

### 3.4 Influence of morphing, speaker, and hormone levels on performance

For B″_D_ model comparisons indicated the best model fit when only morphing level was included as a predictor (see Table 2). This model showed a significant influence only for 2 st Morphing (*t*(192) = −3.67, *p* < 0.001).

**Table 2:** Model comparison for mixed models for response bias B″_D_.

Model	npar	AIC	BIC	*Χ* ^2^	Df	*Pr*(>*Χ*^2^)
**B**″_**D**_ **~ Morph + (1|Proband)**	**6**	**401.456**	**422.820**			
B″_D_ ~ Morph + E + P+ (1|Proband)	8	405.088	433.574	0.367	2	0.832
B″_D_ ~ Morph * E * P+ (1|Proband)	18	407.120	471.213	17.968	10	0.056

Morph = morphing level, E = Estradiol, P = Progesterone, npar = number of parameters. The best fitting model is highlighted in bold.

For RT, the model including the interaction of speaker and morphing level showed the best model fit according to AIC and BIC (see Table 3). All morphing levels influenced RT significantly as well as the interaction of speaker and morphing level for 4 st and 6 st morphing (see Table 4).

**Table 3:** Mixed models for RT.

	npar	AIC	BIC	*Χ* ^2^	Df	*Pr*(>*Χ*^2^)
RT ~ Morph	6	6541.675	6567.198			
RT ~ Morph + Speaker	7	6482.133	6511.91	61.542	1	<0.001
RT ~ Morph + Speaker + E + P	9	6483.31	6521.595	2.823	2	0.244
RT ~ Morph * Speaker	**10**	**6421.501**	**6464.039**	**63.809**	**1**	**<0.001**
RT ~ Morph * Speaker + E + P	12	6422.678	6473.724	2.823	2	0.244
RT ~ Morph * Speaker + E * P	13	6423.59	6478.89	1.088	1	0.297
RT ~ Morph * Speaker * E * P	34	6448.943	6593.573	16.647	21	0.732

Only random effects are depicted in the table, for all models fixed effects were + (1|Proband). Morph = morphing level, Speaker = voice-gender, E = Estradiol, P = Progesterone, npar = number of parameters. The best fitting model is highlighted in bold.

**Table 4:** Model estimates for the model with the best model fit (upper model) and the simplest model including hormones estrogen and progesterone (lower model).

Coefficient	*B*	Df	*t*	*p*
Intercept	1120.734	86.568	40.01	**<0.001**
Morphing 2	76.554	448	4.80	**<0.001**
Morphing 4	126.454	448	7.93	**<0.001**
Morphing 6	214.403	448	13.44	**<0.001**
Speaker	−0.715	448	−0.05	0.964
Morphing 2: Speaker	6.7153	448	0.30	0.766
Morphing 4: Speaker	124.566	448	5.52	**<0.001**
Morphing 6: Speaker	148.031	448	6.56	**<0.001**
Intercept	1085.82	76.250	39.96	**<0.001**
Morphing 2	79.911	451	6.61	**<0.001**
Morphing 4	188.737	451	15.61	**<0.001**
Morphing 6	288.419	451	23.85	**<0.001**
Speaker	69.113	451	8.08	**<0.001**
Estradiol	42.741	62	1.11	0.270
Progesteron	−63.376	62	−1.65	0.104

The number behind Morphing indicates semitones morphed, Speaker = voice-gender. Significant results (*p* < 0.05) are highlighted in bold.

As a comparison, we also took a closer look at the second-best model, which includes estrogen and progesterone. Again, the model showed a significant influence of voice-gender and morphing level, but no influence of either hormone level (see Table 4).

Model estimates for all models can be found in the Supplementary information.

## 4 Discussion

Voice-gender categorization is an important part of everyday social interaction, further influencing mate choice and preferences (Weston et al., 2015). Despite the growing body of evidence, studies investigating the influencing factors on the ability to categorize a voice as masculine or feminine remain scarce. Important influences could be the menstrual cycle phase and female sex hormone profile. Hence, we investigated the associations of menstrual cycle phase with the performance in a voice-gender categorization task using masculine and feminine voices which were morphed toward the other sex, resulting in increasingly ambiguous stimuli.

In contrast to our hypotheses, we did not find any significant differences between cycle phases (follicular vs. luteal) for reaction times (RT), discriminability or response bias. These potential null effects were assessed using different statistical approaches, increasing the likelihood of the null hypothesis being true. In accordance with earlier studies, we observed an increase in RT as well as a decrease in discriminability and correct responses with increasing morphing levels (Smith et al., 2018; Smith et al., 2019). In other words, with increasing ambiguity of the voices’ sex, participants needed more time to decide and were less accurate in doing so. This effect most likely reflects a higher cognitive workload with increasing stimulus ambiguity. Thus, while accuracy was generally high, the paradigm also elicited consistent effects of increasing task difficulty on response times and choices. This may also increase behavioral differences between menstrual cycle phases if present.

Congruent with the findings for reaction times, we did not observe a significant difference between menstrual cycle phases for voice-gender categorization accuracy within our sample. In addition, E and P do not seem to show associations with voice-gender categorization, as investigated by regression models that treated these two variables as continuous predictors. In other studies, estradiol is associated with increased preferences for higher masculinity, both for voices (Feinberg et al., 2006) and faces (Hromatko et al., 2008). Our results did not show such straightforward connection between hormone levels and reaction to male voices. However, it is important to note that fluctuations in progesterone and estradiol are not the only possible explanation for behavioral changes across the cycle. Thus, potential effects could also be driven by an interplay of different sex hormones, which were not investigated in this study such as testosterone, which also influences changes in face preferences (Welling et al., 2007; Niu and Zheng, 2020). Another possible explanation is the influence of other variables affected by the menstrual cycle, such as mood (Cohen et al., 1987; Collins et al., 1985; Pierson et al., 2021) or attention (Pletzer et al., 2017; Thimm et al., 2014). These changes can in turn influence the response behavior.

In addition to the overall effect of morphing level, we found faster RTs for feminine voices compared to masculine voices with higher morphing levels in both cycle phases. This finding also replicates earlier findings on voice-gender categorization, which identified faster reaction times for feminine compared to masculine voices regardless of sex (Junger et al., 2014) or in female participants for morphed voices (Smith et al., 2018; Smith et al., 2019). A possible explanation for faster RTs for female voices could be a higher sensitivity for higher frequencies and specifically female voices in general (Lattner et al., 2005) resulting in a faster reaction to higher pitched voices. As the signal detection theory does not take reaction time into account, this slightly higher sensitivity could accelerate the responses to feminine voices while SDT measures can still be expected to show an other-sex effect for discriminability and response bias.

Regarding response bias, an overall tendency to categorize a voice as male – especially with higher uncertainty - became apparent, thus supporting the idea of an other-sex bias in heterosexual women. The resulting distribution resembles an U shape with smaller biases for original voices and the highest morphing level (6 st) and higher biases for ambiguous voices (2 st and 4 st) with the most pronounced effects for 2 st morphing. While this stronger tendency for 2 st morphing has been found before (Junger et al., 2013, 2014), the underlying mechanism is not yet clear. A possible explanation is the slight ambiguity of the stimuli which increases the effect of the reaction bias. While gender-categorization for unmorphed voices is in most cases an easy task, increased ambiguity heightens the cognitive processing load (Junger et al., 2013). The highest response bias for 2 st morphed voices could reflect the automatic use of heuristics to lighten the processing load (Gigerenzer and Gaissmaier, 2011). As the morphing level increases, overall performance decreases and categorization develops into a more conscious decision process, making the influence of reaction bias less pronounced. Since our sample showed a high variance across all morphing levels, our results must be interpreted with caution.

When looking at the menstrual cycle phases separately, we found a correlation between response bias and reaction time for masculine voices present in the luteal phase, even though there was no significant difference in response bias between phases. A possible explanation could be the influence of cycle phase on hearing sensitivity. Studies on hearing sensitivity could show that sensitivity is higher in the follicular phase than in the luteal phase (Emami et al., 2018; Williamson et al., 2020). A proposed explanation is an enhancing effect of estradiol on hearing which is influenced by the interaction of estradiol with progesterone levels (Williamson et al., 2020). Since there was no interaction of hormone levels in our data, this seems an unlikely explanation for our results. A more likely explanation could be a blunting effect of progesterone on the enhancing effect of estradiol (Al-Mana et al., 2010). Therefore, our results could hint toward a weaker influence of the response bias on reaction times in phases with higher acoustic sensitivity. As we did not assess general hearing sensitivity in this study, this explanation remains speculative and needs to be explored in future studies. Moreover, since interindividual differences in hormone levels are higher than intraindividual fluctuations (Gann et al., 2001), the finding above could be driven by individual hormone profiles that are not strictly linked to cycle phase.

In an exploratory analysis, we looked at regression models in which we examined the potential effect of female sex hormones (P and E) regardless of cycle phase to check for an overall effect of hormone levels. Model comparisons showed that there was no effect of hormones on RT or response bias. Significant predictors of those models were only morphing level and voice-gender. Thus, for the chosen sample, even dimensional models (which have more power compared to ANOVAs) suggest that female sex hormones do not influence sex-voice categorization as measured by RT, and response bias. In line with our previous results, we did not find a clear influence of either cycle phase or sex hormone levels on both accuracy and speed of voice-gender categorization.

Due to the manifold influences on hormonal effects, some limitations of the current study should be considered. For the current sample, we did not differentiate between early and late follicular phase. This may have impacted our results, as estradiol levels change from early to late follicular phase (Sacher et al., 2013). Similarly, we did not account for early, mid, or late luteal phase. However, previous studies show, that differences across cycle phases can be detected using this broader differentiation (Penton-Voak and Perrett, 2000; Johnston et al., 2001; Thimm et al., 2014; Nielsen et al., 2013; Senior et al., 2007). Furthermore, using hormone blood serum levels determined by ECLIAS entails additional uncertainties. Despite their high specificity and common use, a known limitation for all Immunoassays is the potential cross-reactivity with compounds similar to the target hormone, which could potentially influence the calculated blood serum level (Krasowski et al., 2014). Additionally, due to the wider reference range for cycle-phases, misclassification cannot be ruled out, especially in cases where blood serum levels are close to boundaries between cycle phases. Since we only included women in our study, who were rated as being unambiguously within one cycle phase by two independent raters and those ratings had to correspond with the women’s self-reports, we estimate the likelihood of misclassification within our sample as rather small though.

For feasibility reasons, our study used a between-subject design. Within-subject designs are more suitable at detecting changes over the menstrual cycle (Jones et al., 2019; Schmalenberger et al., 2021), so the current study design could have missed subtle changes over the cycle as well as the effect of intraindividual hormone fluctuations. The use of between-subject designs for investigating menstrual-cycle effects is suspected to have a substantial effect on the validity of cycle-phase determination and thus on the statistical power attainable. Referring to estimations by Gonzales and Ferrer (2016), the use of a between-subject design inevitably leads to a higher required sample size, independent of accuracy of cycle-phase determination accuracy. Future studies on voice-gender categorization are therefore advised to use a within-subject design to achieve more robust results, whenever possible. Further methodological precision could be achieved by directly measuring ovulation, using for instance LH tests in a standardized way as recommended by Blake et al. (2016).

Besides, additional hormones may also be considered in their interaction possibly influencing voice-gender categorization. For example, testosterone and thyroid hormones both influence cochlear development and hearing and may be investigated in future studies on voice-gender categorization tasks (Frisina et al., 2021). This is especially important considering that for feasibility reasons we could not measure all participants at the same time of day. Since all sex hormones present a specific circadian rhythm (Dabbs and de La Rue, 1991; Rahman et al., 2019), potential effects of time of day on the measured blood-serum level as well as potential interactions of progesterone and estradiol with the aforementioned additional hormones cannot be included in the analyses.

Looking at the manifold influences involved in analyses of hormonal effects, the sample size used in our study was likely not sufficient after all. Each factor brings a certain degree of variance into the analyses that cannot be accounted for in a regular power-analysis for between-subject comparisons. Therefore, sample size calculations should be based on methods considering the specifics of menstrual-cycle research (e.g., Gangestad et al., 2016; Gonzales and Ferrer, 2016; Schmalenberger et al., 2021). The shortcoming of not taking those specifics into account likely lead to a decrease in statistical power and therefore to a higher probability of accepting the null hypothesis while it was not true. Thus, we strongly recommend replicating the study design using a higher number of participants as well as a within-subjects design to increase statistical power.

As the originally spoken words were manipulated for the paradigm, especially the higher morphing levels sounded less natural, which could lead to diminished ecological validity and thus to a weakened influence of mechanisms important for mate choice. Additionally, stimuli were controlled for and changed in F0, but not in degree of breathiness, which could further influence voice-gender perception (Whitling et al., 2023). Nevertheless, considering that the exact same paradigm was used multiple times before and robustly showed effects of both gender and sexual orientation (Junger et al., 2013; Junger et al., 2014; Smith et al., 2018; Smith et al., 2019), we do assume this effect does not exert major influence on our results, especially in view of various studies successfully using mechanically morphed voices to examine even more complex social cues such as perceived dominance (e.g., Brown et al., 1973; Feinberg et al., 2005; Wang et al., 2018).

As mentioned earlier, there is a growing number of studies that do not find clear influences of menstrual cycle phase on female behavior such as mating behavior (Stern et al., 2021; Stern et al., 2022; Holzleitner et al., 2022). In two different studies, Harris (2011, 2013) tested different methods commonly used in studies on menstrual cycle effects on attractiveness ratings for male faces but did not find any influence of cycle phase on the participants’ ratings. Likewise, eye-tracking studies testing the influence of hormonal changes on attractiveness ratings for male faces and bodies did not find associations with cycle phase either (Garza and Byrd-Craven, 2019; Garza and Byrd-Craven, 2023). Furthermore, meta-analyses on preference shifts across sensory modalities (i.e., faces, voices and scent) showed only a few effects, which were likely due to an imprecise definition of the fertile phase (Wood, 2016; Wood et al., 2014). Nevertheless, there is still an ongoing debate about the presence or absence as well as the magnitude of the influence of menstrual cycle phase on behavior, as other studies using similar tasks still find large effects on similar questions (Jones et al., 2008). Additional disagreement arises through divergent interpretations of the increasing number of null effects (Wood, 2016; Gangestad, 2016).

Suspected reasons for an overestimation of a cycle dependent shift in attractiveness ratings are a high publication bias as well as a high degree of freedom when it comes to researchers’ decisions in sampling, study design and methods. Thus, confirmatory hypothesis testing can lead to arbitrary exclusion of participants, a broader definition of the fertile window and an inconsistent choice of moderators across analyses to achieve significant results (Wood et al., 2014; Harris, 2013). However, some of the studies presenting null effects show similar methodological shortcomings and are faced with criticism concerning sample sizes and statistical power (e.g., DeBruine et al., 2010) and thus regarding the interpretation of null effects (Gildersleeve et al., 2013). Moreover, studies seem to show only sparse evidence for the evolutionary perspective of female mate selection being driven by increasing chances for optimal offspring (Harris, 2013). One possible explanation is the negligence of socio-economic and sociosexual influences on mate choice which can be expected to play a stronger role than potential hormonal influences (Wood et al., 2014; Albert et al., 2018). Taken together, our results fall in line with the accumulating findings, that the associations of menstrual cycle phase with female (mating) behavior found in earlier studies might be a less robust effect than originally assumed, thus contributing to the ongoing debate about factors that might influence the complex interplay of sex hormones and behavior.

In conclusion, in this first study on associations of menstrual cycle phase with performance in voice-gender categorization, we did not find any significant differences between cycle phases for discriminability, response bias or reaction time in a between-subject design. Investigating effects regardless of cycle phase also did not show any significant associations of hormone levels and performance. Therefore, there might be no straightforward association between menstrual cycle phase or sex hormone level and voice-gender perception, supporting a growing body of literature reporting no or only subtle effects of menstrual cycle phase on female mating behavior. However, interpretation of results is impeded by multiple factors relevant not only in analyses, but also in study design for menstrual cycle research, such as sampling method and cycle phase confirmation. Keeping those drawbacks in mind, our study seems to support the idea, that earlier studies on the matter might have overestimated the influence of sex hormone fluctuations on women’s behavior. Further research in this line of research is needed to shed light on the interplay of hormones, socioeconomic factors, and behavior. The diverging results thus far highlight the importance of standardized best practices guidelines for sampling, sample size and interpretation of results for menstrual cycle research to minimize confounding factors and allow for a higher comparability of results across studies.

## Data Availability

The original contributions presented in the study are included in the article/Supplementary material, further inquiries can be directed to the corresponding author.

## Footnotes

^1^ https://osf.io/dteyn
