# Supplementary material for: Assessing the association between menstrual cycle phase and voice-gender categorization: no robust evidence for an association
Source: Front Psychol. 2025 Apr 2;16:1531021. doi: 10.3389/fpsyg.2025.1531021 (PMC12031663; doi:10.3389/fpsyg.2025.1531021)
Supplement: Supplementary file 1 [file Supplementary_file_1.pdf]

Supplementary Information - Assessing the association between menstrual cycle phase and voice-gender categorization:no robust evidence for an association

Supplementary Information 1: Description of cycle phase determination

In addition to forward counting of cycle days based on women's self-report, cycle phase was determined for each woman based on reference values for progesterone (P) and estradiol (E) in blood. To avoid a biased decision, this was not only performed by the investigator, but also by two additional raters regarding the pre-existing datasets and by one additional rater for the newly collected data. Raters were not involved in data collection or analyses or any other part of this study. For the cycle phase determination, raters were only given the reference values for the different cycle phases and the hormone values for each woman. Only women who could be clearly categorized according to these values were included in the analyses.

Supplementary information 2: Pairwise comparisons and model estimates for mixed models

Table 1. pairwise post-hoc tests for differences in RT for different morphing levels for all trials. Results are Bonferroni corrected for multiple testing.

Morphing	*n*	*t*	*df*	*p*
0 st – 2 st	130	-10.077	129	$3.4e^{-17}$ *
0 st – 4 st	130	-16.616	129	$4.48e^{-33}$ *
0 st – 6 st	130	-19.083	129	$1.33e^{-38}$ *
2 st – 4 st	130	-11.138	129	$7.92e^{-20}$ *
2 st – 6 st	130	-15.317	129	$4.9e^{-30}$ *
4 st – 6 st	130	-9.479	129	$1.01e^{-15}$ *

Table 2. pairwise post-hoc tests for differences in RT for different morphing levels for correct trials. Results are Bonferroni corrected for multiple testing.

Morphing	*n*	*t*	*df*	*p*
0 st – 2 st	130	-9.549	129	$6.84e^{-16}$ *
0 st – 4 st	130	-14.878	129	$5.43e^{-29}$ *
0 st – 6 st	130	-19.687	129	$6.6e^{-40}$ *
2 st – 4 st	130	-9.348	129	$2.12e^{-15}$ *
2 st – 6 st	130	-17.298	129	$1.23e^{-34}$ *
4 st – 6 st	130	-12.574	129	$2.19e^{-23}$ *

Table 3. pairwise post-hoc test for differences in A' for different morphing levels over both groups. Results are Bonferroni corrected for multiple testing.

Morphing	*n*	*t*	*df*	*p.adj*
0 st – 2 st	66	11.959	65	$2{,}7e^{-17}$ *
0 st – 4 st	66	17.848	65	$5{,}71e^{-26}$ *
0 st – 6 st	66	22.943	65	$4{,}27e^{-32}$ *
2 st – 4 st	66	14.303	65	$5{,}66e^{-21}$ *
2 st – 6 st	66	21.896	65	$6{,}3e^{-31}$ *
4 st – 6 st	66	19.820	65	$1{,}78e^{-28}$ *

Table 4. pairwise post-hoc test for differences in B"D for different morphing levels over both groups. Results are Bonferroni corrected for multiple testing.

Morphing	*n*	*t*	*df*	*p.adj*
0 st – 2 st	66	2.788	64	0.042 *
0 st – 4 st	66	1.602	64	0.684
0 st – 6 st	66	-1.150	64	1
2 st – 4 st	66	-1.922	65	0.354
2 st – 6 st	66	-4.752	65	$6.90e^{-05}$ *
4 st – 6 st	66	-5.464	65	$4.73e^{-06}$ *

Table 5: Model estimates for all mixed models for response bias B''_D.

		Estimate	Df	*t*	*p*
1	Intercept	-0.102	129.48	-1.31	.192
	Morph 2	-0.264	192	-3.67	**<.001**
	Morph 4	-0.119	192	-1.66	.098
	Morph 6	0.096	192	1.34	.183
2	Intercept	-0.102	124.06	-1.30	.196
	Morph 2	-0.264	192	-3.67	**<.001**
	Morph 4	-0.119	192	-1.66	.098
	Morph 6	0.096	192	1.34	.183
	Estradiol	0.053	62	0.55	.587
	Progesterone	-0.054	62	-0.56	.578
3	Intercept	-0.182	118.45	-1.77	0.080
	Morph 2	-0.169	183	-1.82	0.070
	Morph 4	0.050	183	0.54	0.591
	Morph 6	0.249	183	2.68	**0.008**
	Estradiol	-0.055	118.45	-0.45	0.654
	Progesterone	-0.046	118.45	-0.38	0.707
	Morph 2:Estradiol	0.005	183	0.05	0.964
	Morph 4:Estradiol	0.295	183	2.68	**0.008**
	Morph 6:Estradiol	0.182	183	1.66	0.100
	Morph 2:Progesterone	0.126	183	1.16	0.248
	Morph 4:Progesterone	-0.092	183	-0.84	0.400
	Morph 6:Progesterone	-0.022	183	-0.12	0.842
	Estradiol:Progesterone	0.110	118.45	1.20	0.234
	Morph 2:Estradiol:Progesterone	-0.128	183	-1.55	0.122
	Morph 4:Estradiol:Progesterone	-0.230	183	-2.79	**0.006**
	Morph 6:Estradiol:Progesterone	-0.207	183	-2.51	0.013

Note: Model 1: bppd ~ Morph + (1|Proband), Model 2: bppd ~ Morph + Estradiol + Progesterone + (1|Proband), Model 3: bppd ~ Morph*Estradiol*Progesterone + (1|Proband). The number behind Morph indicates semitones morphed

Table 6: Model estimates for all mixed models for reaction time.

		B	Df	*t*	*p*
1	Intercept	1120.38	76.37	41.30	**<.001**
	Morph 2	79.91	452	6.18	**<.001**
	Morph 4	188.74	452	14.60	**<.001**
	Morph 6	288.42	452	22.31	**<.001**
2	Intercept	1085.82	78.53	39.75	**<.001**
	Morph 2	79.911	451	6.61	**<.001**
	Morph 4	188.737	451	15.61	**<.001**
	Morph 6	288.419	451	23.85	**<.001**
	Speaker m	69.113	451	8.08	**<.001**
3	Intercept	1120.734	86.57	40.01	**<.001**
	Morphing 2	76.554	448	4.80	**<.001**

	Morphing 4	126.454	448	7.93	**<.001**
	Morphing 6	214.403	448	13.44	**<.001**
	Speaker m	-0.715	448	-0.05	0.964
	Morphing 2: speaker m	6.7153	448	0.30	0.766
	Morphing 4: speaker m	124.566	448	5.52	**<.001**
	Morphing 6: speaker m	148.031	448	6.56	**<.001**
4	Intercept	1085.82	76.25	39.96	**<.001**
	Morphing 2	79.911	451	6.61	**<.001**
	Morphing 4	188.737	451	15.61	**<.001**
	Morphing 6	288.419	451	23.85	**<.001**
	Speaker m	69.113	451	8.08	**<.001**
	Estradiol	42.741	62	1.11	.270
	Progesterone	-63.376	62	-1.65	.104
5	Intercept	1120.734	84.15	40.21	**<.001**
	Morphing 2	76.554	448	4.80	**<.001**
	Morphing 4	126.454	448	7.93	**<.001**
	Morphing 6	214.403	448	13.44	**<.001**
	Speaker m	-0.715	448	-0.05	.964
	Estradiol	42.741	62	1.11	.270
	Progesterone	-63.376	62	-1.65	.104
	Morphing 2: speaker m	6.715	448	0.30	.766
	Morphing 4: speaker m	124.567	448	5.52	**<.001**
	Morphing 6: speaker m	148.031	448	6.56	**<.001**
6	Intercept	1143.361	73.15	32.04	**<.001**
	Morphing 2	76.554	448	4.80	**<.001**
	Morphing 4	126.454	448	7.93	**<.001**
	Morphing 6	214.403	448	13.44	**<.001**
	Speaker m	-0.7153	448	-0.05	.9670
	Progesterone	-52.337	61	-1.31	.194
	Estradiol	55.121	61	1.37	.176
	Morphing 2: speaker m	6.715	448	0.30	.766
	Morphing 4: speaker m	124.567	448	5.52	**<.001**
	Morphing 6: speaker m	148.031	448	6.56	**<.001**
	Progesterone	-30.639	61	-1.02	.314
7	Intercept	1137.1	83.05	30.84	**<.001**
	Morph 2	71.807	427	3.37	**.001**
	Morph 4	136.954	427	6.46	**<.001**
	Morph 6	206.750	427	9.75	**<.001**
	Speaker m	5.104	427	0.24	.810
	Progesterone	-49.842	83.05	-1.16	.251
	Estradiol	29.461	83.05	0.68	.501
	Morph 2:speaker m	16.844	427	0.56	.575
	Morph 4:speaker m	127.498	427	4.25	**<.001**
	Morph 6:speaker m	165.581	427	5.52	**<.001**
	Morph 2:Progesterone	3.893	427	0.16	.875
	Morph 4:Progesterone	5.350	427	0.22	.829

Morph 6:Progesterone	2.353	427	0.10	.924
Speaker m:Progesterone	-8.088	427	-0.32	.747
Morph 2:Estradiol	22.288	427	0.89	.374
Morph 4:Estradiol	22.041	427	0.88	.379
Morph 6:Estradiol	10.777	427	0.43	.667
Speaker m:Estradiol	33.963	427	1.36	.176
Progesterone:Estradiol	-22.161	83.05	-0.68	.500
Morph 2:speaker m:Progesterone	9.945	427	0.28	.777
Morph 4:speaker m:Progesterone	-10.877	427	-0.31	.757
Morph 6:speaker m:Progesterone	-10.181	427	-0.29	.772
Morph 2:speaker m:Estradiol	-20.331	427	-0.57	.566
Morph 4:speaker m:Estradiol	-19.535	427	-0.55	.582
Morph 6:speaker m:Estradiol	-0.917	427	-0.03	.979
Morph 2:Progesterone:Estradiol	6.427	427	0.34	.732
Morph 4:Progesterone:Estradiol	-14.218	427	-0.76	.449
Morph 6:Progesterone:Estradiol	10.364	427	0.55	.581
Speaker m:Progesterone:Estradiol	-7.880	427	-0.42	.675
Morph 2:speaker m:Progesterone:Estradiol	-13.716	427	-0.52	.606
Morph 4:speaker m:Progesterone:Estradiol	-3.970	427	-0.15	.881
Morph 6:speaker m:Progesterone:Estradiol	-23.766	427	-0.90	.371
Progesterone:Estradiol	1137.1	83.05	30.84	**<.001**
Morph 2:speaker m:Progesterone	71.807	427	3.39	**.001**
Morph 4:speaker m:Progesterone	136.954	427	6.46	**<.001**
Morph 6:speaker m:Progesterone	206.750	427	9.75	**<.001**
Morph 2:speaker m:Estradiol	5.104	427	0.24	.810
Morph 4:speaker m:Estradiol	-49.842	83.05	-1.16	.251
Morph 6:speaker m:Estradiol	29.461	83.05	0.68	.501

Note: Modell 1: RT ~ morph + (1|Proband),
Model 2: RT ~ morph + speaker + (1|Proband),
Model 3: RT ~ morph * speaker + (1|Proband)
Model 4: RT ~ morph + speaker + Estradiol + Progesterone + (1|Proband)
Model 5: RT ~ morph * speaker + Estradiol + Progesterone +(1|Proband)
Modell 6: RT ~ morph * speaker + Estradiol * Progesterone +(1|Proband)
Model 7: RT ~ morph * speaker * Estradiol * Progesterone +(1|Proband)
The number behind Morphing indicates semitones morphed, Speaker = voice gender.
